# Knowledge about diabetes mellitus and its associated factors among diabetic outpatients at Muhimbili National Hospital in Tanzania

**DOI:** 10.11604/pamj.2023.45.3.33143

**Published:** 2023-05-03

**Authors:** Christine Luambano, Bertha Mwinuka, Rogate Phinias Ibrahim, Godfrey Kacholi

**Affiliations:** 1 Department of Health Systems Management, School of Public Administration and Management, Mzumbe University, Morogoro, Tanzania,; 2 Centre of Excellence in Health Monitoring and Evaluation, Mzumbe University, Morogoro, Tanzania

**Keywords:** Knowledge, diabetes mellitus, socio-demographic factors, Muhimbili National Hospital

## Abstract

Diabetes mellitus is emerging as one of the major public health threats that contributed to 2% of all deaths in Tanzania in 2016. Although adequate knowledge related to diabetes mellitus is associated with early case detection, prevention, and minimization of health complications and socioeconomic-related consequences, there is less evidence about the adequacy of the community´s knowledge of diabetes in Tanzania. This study aimed to determine knowledge about diabetes mellitus and its associated factors among diabetic outpatients. A cross-sectional study was conducted among 220 diabetic outpatients aged 18 years and above at Muhimbili national hospital in Tanzania between February and April 2017. Data were collected using a structured pretested questionnaire and were entered into Microsoft Excel and exported to SPSS Version 20 for analysis. Bivariate and multivariate logistic regression was used to determine the predictive variables. The significance of independent variables was declared at a 95% confidence level and p-value < 0.05. A total of 137 (64.01%) of the participants had adequate knowledge about diabetes mellitus. The majority (86.9% and 85.1%) reported having adequate knowledge of complications of diabetes and treatment options for diabetes respectively. The least level of knowledge reported was on signs and symptoms (48.6%) and type of diabetes (32.7%). The majority (54%) cited health facilities as the most common sources of information related to diabetes. Both bivariate and multivariate logistic regression analyses showed that there was a statistical association between knowledge related to diabetes and the level of education of study participants. The overall level of knowledge of participants about diabetes mellitus was adequate, with a low level of knowledge related to signs and symptoms of diabetes, and type of diabetes. Health facilities were the most common sources of information related to diabetes. Policy and decision-makers and health care providers should take collective action to improve community knowledge about diabetes. Health education related to diabetes should be integrated into the educational curriculum at all levels in Tanzania, which would massively increase awareness of diabetes.

## Introduction

Diabetes Mellitus is one of the serious and devastating chronic diseases of public health concern that affects the lives and well-being of individuals across the globe [1]. The World Health Organisation (WHO) estimated that diabetes mellitus was responsible for almost 1.5 million deaths, with at least 420 million people (6%) affected by diabetes mellitus globally in 2019 [2]. The prevalence of diabetes mellitus has been increasing rapidly, especially in low- and middle-income countries [3]; as a result of ongoing rapid increased urbanization that fuels changes in unhealthy lifestyles [4]. According to the International Diabetes Federation (IDF), diabetes mellitus is estimated to increase from 382 million people in 2013 to 592 million by 2035 globally [4]. Generally, the impact of diabetes mellitus includes damaging of heart, blood vessels, eyes, kidneys, and nerves [5], which is often associated with obesity and physical inactivity [6]. In sub-Saharan Africa (SSA), at least 12 million people suffered from diabetes mellitus, and approximately 330,000 people died due to diabetes mellitus in 2020 [7]. This burden has been increasing from time to time and the estimates show that approximately 40.7 million people will be affected by diabetes mellitus by 2045, from 15.9 million people in 2017 [8]. Tanzania is among the SSA countries experiencing a double burden of communicable and non-communicable diseases [9]. Diabetes mellitus as one of the non-communicable diseases contributed to 2% of all deaths in Tanzania in 2016 [10]. Given the increased urbanization, greatest increases in the older population, lifestyle changes as well as unhealthy diets and lack of physical activity, the prevalence and number of deaths due to diabetes mellitus are expected to increase rapidly in the next decades in Tanzania [11]. This is likely to cause more substantial economic harm to diabetic patients and their respective households while simultaneously stretching the already overburdened health care systems of Tanzania.

In recognition of this challenge, the Government of Tanzanian through its Ministry of Health, Community Development, Gender, Elderly and Children (MoHCDGEC) developed a National Non-communicable Diseases Prevention and Control Strategy to ensure improved access, availability, and affordability of quality non-communicable services in the country [12]. These efforts have been implemented parallel with the program of constructing and rehabilitating health facilities, increasing the number of skilled health workers, increasing the supply of drugs and diagnostic equipment, and health promotion at all levels throughout the country [13]. However, the prevalence seems to continue increasing. Adequate knowledge related to diabetes mellitus is of paramount importance not only in early case detection and prevention but also in minimizing health complications and socioeconomic-related consequences [14]. Studies have shown that public health knowledge on modifiable factors such as a healthy diet and physical activeness can significantly delay premature deaths from diabetes mellitus [15]. Several studies conducted in low and middle-income countries have shown a good level of knowledge about diabetes mellitus can help in early case detection, prevention, and minimization of the adverse consequences [16-18]. Furthermore, previous studies have shown socio-demographic factors such as age, gender, level of education, history of diabetes mellitus in the family were associated with adequate knowledge related to the disease [19-22]. There is limited evidence on the level of knowledge and associated factors among diabetic patients in Tanzania. Therefore, this study aimed to assess knowledge about diabetes mellitus and its associated factors among diabetic outpatients at Muhimbili National Hospital in Tanzania, the results of this study are envisioned to provide relevant information on the current knowledge-related status that will inform the policymakers and stakeholders to design appropriate interventions that will stimulate both early detection and appropriate management of diabetes mellitus within and outside the country.

## Methods

**Study design and setting:** this was a descriptive cross-sectional study conducted among outpatients at the Muhimbili National Hospital diabetes clinic. The hospital is located in Dar es Salaam, the largest most commercial city in Tanzania. The hospital serves as a national referral hospital while simultaneously functioning as a teaching and research facility for the Muhimbili University of Health and Allied Sciences. It offers specialized care across the spectrum of clinical medicine. The hospital has both adult and pediatric diabetes clinics. The study was conducted between February and April 2017.

**Sampling and study population:** the study enrolled a total of 220 outpatients from an estimation of 5760 diabetes outpatients´ attendance per year. Patients who attended the clinic within one month of data collection were consecutively included in the sample. Outpatients attending diabetes mellitus services aged 18 years or above were included in the study based on their consent. Enrolled study participants were either newly diagnosed diabetes patients or patients who were continuing with diabetes mellitus treatments. Patients with other illnesses besides diabetes mellitus were excluded from the study.

**Data collection methods and instruments:** we used a semi-structured questionnaire to collect data from outpatients in the diabetes clinic at Muhimbili National Hospital. The development of the questionnaire was informed by relevant literature [15-22]. The questionnaire consisted of two parts. The first part aimed to collect socio-demographic characteristics of study participants such as age, gender, occupation, education level, marital status, area of residence, and years with diabetes while the second part aimed to collect information related to participants´ level of knowledge related to diabetes mellitus, and sources of diabetes information. The scoring of the knowledge questions was coded as (yes) answer =1 and (no) answer=0. The questionnaire was prepared in English and translated into Swahili (a language commonly used by all Tanzanians). The translation process involved two language experts to ensure the accuracy of the translation. Exit interviews were conducted by the first author in the outpatient areas.

**Data quality control and assurance:** the questionnaire was pretested among 25 outpatients at the Morogoro Regional Referral Hospital two weeks before the commencement of the actual study. The pre-testing aimed to check for the consistency, accuracy, and relevance of the questions. The questionnaire was revised based on the responses to the pretest. The analysis of the pre-tested data was excluded from the overall results of this study. The filled questionnaires were checked daily by the first author and reviewed by the second author weekly to ensure completeness and consistency. A double data entry technique was used.

**Variable measurement:** the level of knowledge of diabetes mellitus was taken as the dependent variable. The independent variables include socio-demographic characteristics of participants (age, gender, occupation, education level, marital status, area of residence, and years with diabetes mellitus).

**Data analysis:** the collected data were entered into MS Excel to be checked and cleaned before being exported into SPSS version 20 for analysis. Descriptive statistics such as frequency and percentage were computed. A composite knowledge variable from knowledge questions (knowledge on types, causes, treatment, causes, symptoms, and complications of diabetes mellitus) was created, assigning numbers 1 and 0 for adequate and inadequate knowledge respectively for each of the knowledge questions. Bivariate and multivariate logistic regression was used to determine the predictive variables. The significance of independent variables was declared at a 95% confidence level and p-value < 0.05.

**Ethical considerations:** the protocol was reviewed and permission to collect data was granted by the leadership of Muhimbili Diabetic Clinic with reference MNH/TRC/Research/2017/014. Before data collection, every participant was informed about the nature, purpose, and procedures of the study. Participants were asked to sign the consent form for their acceptance to participate in the study. Participants were given the freedom to either answer specific questions or withdraw from the study at any time. To ensure anonymity, identity numbers rather than names were used to represent the study participants.

## Results

**Socio-demographic characteristics of study participants:** a total of 214 out of 220 enrolled participants from the Muhimbili Diabetic Clinic participated in this study. The mean age was 37 years with equal gender distribution among the participants. More than three-quarters (84.6%) of participants were employed. Almost half of the participants had either a primary education (45.3%) or no formal education (7.5%), and the majority were married (53.7%). The majority (68.7%) of participants lived in Dar es Salaam and 66.8% had diabetes mellitus for more than five years (Table 1).

**Table 1:** socio-demographic of study participants (n= 214)

Characteristics	Frequency	Percentage
**Age in years**		
≤37	70	32.7
>37	144	67.3
**Sex**		
Male	107	50.0
Female	107	50.0
**Occupation**		
None employed	33	15.4
Employed	181	84.6
**Education level**		
No formal	16	7.5
Primary	97	45.3
Post-primary	101	47.2
**Marital status**		
Single	118	55.1
Married	96	44.9
**Years with diabetes mellitus**		
≤5	24	11.2
>5	190	88.8
**Place of residence**		
Outside DSM	67	31.3
Live in DSM	147	68.7

**Figure 1:**
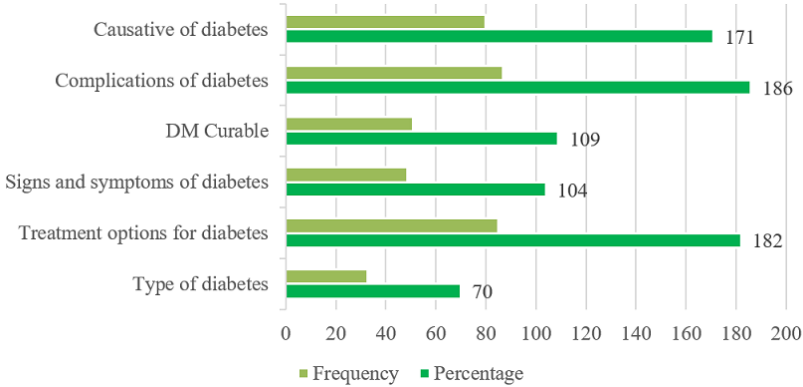
levels of knowledge related to diabetes

**Knowledge score related to diabetes mellitus:** a total of 137 (64.01%) of the participants were knowledgeable about diabetes mellitus. The majority of study participants (n = 186; 86.9%) had knowledge on complications of diabetes mellitus, followed by knowledge on treatment options for diabetes (n= 182; 85.1%) and causatives of diabetes (n=171; 79.9%). The least level of knowledge was on signs and symptoms of diabetes mellitus (n= 104; 48.6%) and type of diabetes mellitus (n= 70; 32.7%) (Figure 1).

**Figure 2:**
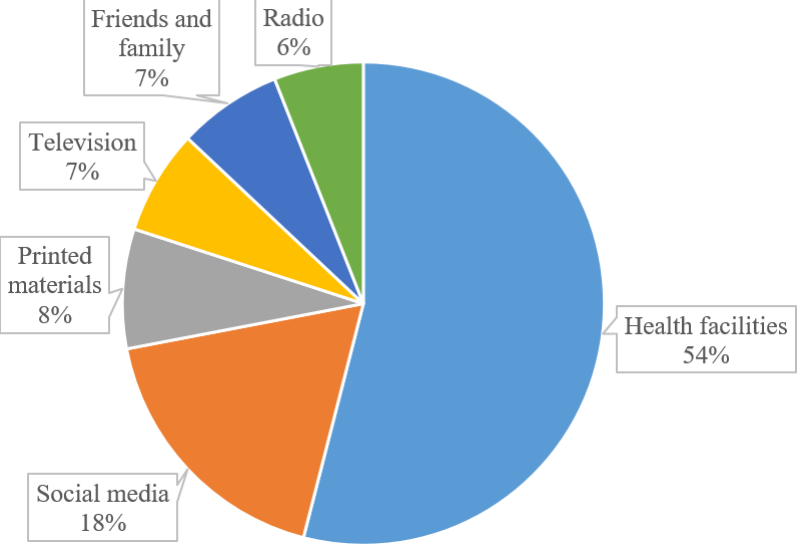
potential sources of information related to diabetes

**Potential sources of information related to diabetes mellitus:** health facilities were reported by the majority (54%) of study participants as the most common sources of information related to diabetes mellitus followed by social media (18%) (Figure 2).

**Associations between knowledge related to diabetes mellitus and socio-demographic characteristics:** both bivariate and multivariate logistic regression analyses showed that there was a statistical association between knowledge related to diabetes mellitus and socio-demographic characteristics. In bivariate logistic regression analysis, education (primary) (OR 4.08; 95% CI 1.31-12.70, p=0.0154); education (post-primary) (OR 6.03; 95% CI 1.92-18.95, p=0.0021) showed an increased odds of knowledge for diabetes. In multivariate logistic regression analysis, education (primary) (OR 3.86; 95% CI 1.23-12.10, p=0.0205); education (post-primary) (OR 5.73; 95% CI 1.81-18.11, p=0.0029) showed a decrease odd of knowledge for diabetes mellitus. However, there was no statistical association between age, sex, occupation, marital status, years lived with diabetes mellitus, and place of residence of participants with their knowledge related to diabetes mellitus (Table 2).

**Table 2:** associations between knowledge related to diabetes and socio-demographic characteristics

Characteristics	Inadequate	Adequate	Unadjusted		Adjusted	
	N (%)	N (%)	OR[95%CI]	p-value	OR[95%CI]	p-value
**Age**						
≤37	22(31.43)	48(68.57)	Ref		Ref	
>37	50(34.72)	94(65.28)	0.86[0.47, 1.59]	0.6325	0.95[0.48, 1.88]	0.8775
**Sex**						
Male	33(30.84)	74(69.16)	Ref		Ref	
Female	39(36.45)	68(63.55)	0.78[0.44, 1.37]	0.3858	0.84[0.46, 1.53]	0.5648
**Occupation**						
None employed	11(33.33)	22(66.67)	Ref		Ref	
Employed	61(33.70)	120(66.30)	0.98[0.45, 2.16]	0.9672	1.39[0.54, 3.64]	0.4951
**Education level**						
No formal	11(68.75)	5(31.25)	Ref		Ref	
Primary	34(35.05)	63(64.95)	4.08[1.31, 12.70]	0.0154	3.52[1.07, 11.51]	0.0377
Post-primary	27(26.73)	74(73.27)	6.03[1.92, 18.95]	0.0021	5.65[1.62, 19.68]	0.0065
**Marital status**						
Single	35(29.66)	83(70.34)	Ref		Ref	
Married	37(38.54)	59(61.46)	0.67[0.38, 1.19]	0.1724	0.77[0.39, 1.48]	0.4287
**Years with diabetes**						
≤5	6(25.00)	18(75.00)	Ref		Ref	
>5	66(34.74)	124(65.26)	0.63[0.24, 1.65]	0.3451	0.73[0.26, 2.02]	0.5435
**Place of residence**						
Outside DSM	26(38.81)	41(61.19)	Ref		Ref	
Live in DSM	46(31.29)	101(68.71)	1.39[0.76, 2.54]	0.2816	1.31[0.68, 2.49]	0.4194

## Discussion

This study determined the level of knowledge related to diabetes mellitus, explored potential sources of diabetes mellitus information and the associations between knowledge related to diabetes mellitus and socio-demographic characteristics in a sample of outpatients attending a diabetic clinic at Muhimbili National Hospital in Tanzania. Our analysis showed that 64.01% of the participants were knowledgeable about diabetes mellitus. The level of knowledge related to diabetes mellitus reported in the current study is higher than those reported in previous studies of similar socio-demographics in sub-Saharan Africa [20-22]. Their high level of knowledge could be attributed to the reasons that the majority were diagnosed and lived with diabetes mellitus for more than five years. Studies conducted in South Africa showed that patients with diabetes mellitus were more likely to be knowledgeable about the disease compared to those without the disease [23].

Over 86% of patients with diabetes mellitus in this study were knowledgeable about the complications of the disease. Previous studies from both developed and developing countries have shown that knowledge related to diabetes mellitus complications is often good among patients with the same disease [23-26]. This could be attributed to the duration of living with diabetes mellitus and the types of treatments given as well as experience of having complications. Our study did not explore patients´ knowledge of severity levels of diabetes mellitus complications. In addition, the vast majority (85.1%) of patients demonstrated good knowledge of treatment options for the disease. This result is similar to the results of the study conducted in Ghana that found most of the diabetes mellitus outpatients knew about the available treatment options for the disease [27]. Although participants reported knowing diabetes mellitus treatment options, we did not explore their levels of knowledge on treatment options such as using insulin to manage their sugar levels as well as diet and regular physical exercises.

Knowledge regarding the signs and symptoms of a particular disease among people within the community is compulsory for its proper management and to appropriately control the further spread of such disease within and outside the context [28]. The current study indicated that the level of knowledge on signs and symptoms of diabetes mellitus among patients was seemingly low. This result is contrary to the results of the cross-sectional study conducted in Uganda among university students which found knowledge with regard to symptoms of diabetes mellitus was adequate [28]. Likewise, poor knowledge related to types of diabetes was scarcely reported by 32.7% of patients. This indicates that although the patients were fairly educated but were unaware of the types of diabetes mellitus. Previous studies have shown that knowledge related to types of diabetes mellitus across the population is important and can help the general population overcome the disease and its associated complications [29].

Results from the current study revealed that patients had good knowledge of the general information related to diabetes mellitus. Health facilities were mostly reported as the main sources of information among diabetes mellitus patients. This result is not surprising because previous studies have reported that often patients prefer to receive information from healthcare providers, whose information is mostly accurate, trusted, and reliable [30-32]. Although social media is considered a powerful channel of communication and plays a very important role in today's life, few patients reported it as their source of information. In 2020, the telecom statistics published by the Tanzania Communications Regulatory Authority (TCRA) estimated internet users reached 28.5% [33]. Although the internet is considered to offer more privacy while simultaneously providing more accessible information as compared to face-to-face healthcare providers, it appears practices of seeking online health information are poor. This suggests that further study is urgently needed to respond to this paradox.

Although no statistical association between the ages, sex, occupation, marital status, years lived with diabetes mellitus, and place of residence of participants, the results showed a significant association of knowledge related to diabetes with the educational levels of patients. The participants who had primary and post-primary education were more likely to have good knowledge related to diabetes mellitus. Literature has shown that people with a considerable level of education are more likely to have adequate knowledge of diabetes mellitus [34], which is the case in the current study. This may be due to the amount of awareness accumulated through both formal and informal training, which enables patients to become well-informed about the disease.

### Strengths and limitations

This study has multiple strengths: first, the study enrolled participants from the city (Dar es Salaam) and those outside the city accessing diabetes mellitus at the hospital placed at the tertiary level of health service delivery in Tanzania. Second, the study was conducted in a hospital that attracts outpatients with a diversity of socio-demographic factors. Third, the questionnaire used was adapted from the previous literature, with slight modifications. However, the study findings should be interpreted with caution because the study was conducted in one hospital and therefore reliability and generalizability of the results could be limited.

## Conclusion

The study participants had adequate knowledge related to diabetes mellitus and demonstrated a high level of knowledge of the treatment and complications of diabetes mellitus. Less knowledge of the types, signs, and symptoms of diabetes appeared the most common challenge. Health facilities were the main sources of diabetes-related information. The present study showed that social media were not adequately utilized to access diabetes mellitus-related information. The level of education was significantly associated with knowledge related to diabetes. Results of this study highlighted the need for utilizing social media with well-designed content to increase knowledge about diabetes that might promote a healthy lifestyle. It is surprising in this study that participants were aware of complications of diabetes mellitus but less knowledgeable of signs and symptoms and types of diabetes mellitus. Therefore, a further qualitative study needs to be conducted to confirm this observation and the potential explanations.
